# ‘*Candidatus* Xiphinematincola pachtaicus' gen. nov., sp. nov., an endosymbiotic bacterium associated with nematode species of the genus *Xiphinema* (Nematoda, Longidoridae)

**DOI:** 10.1099/ijsem.0.004888

**Published:** 2021-07-21

**Authors:** Juan E. Palomares-Rius, Carlos Gutiérrez-Gutiérrez, Manuel Mota, Wim Bert, Myriam Claeys, Vladimir V. Yushin, Natalia E. Suzina, Elena V. Ariskina, Lyudmila I. Evtushenko, Sergei A. Subbotin, Pablo Castillo

**Affiliations:** ^1^​ Institute for Sustainable Agriculture (IAS), Spanish National Research Council (CSIC), Avenida Menéndez Pidal s/n, 14004 Córdoba, Campus de Excelencia Internacional Agroalimentario, ceiA3, Spain; ^2^​ NemaLab, MED – Mediterranean Institute for Agriculture, Environment and Development, Institute for Advanced Studies and Research, Universidade de Évora, Pólo da Mitra, Ap. 94, 7006-554 Évora, Portugal; ^3^​ Nematology Research Unit, Department of Biology, Ghent University, K.L. Ledeganckstraat 35, 9000 Ghent, Belgium; ^4^​ A.V. Zhirmunsky National Scientiﬁc Center of Marine Biology, Far Eastern Branch, Russian Academy of Sciences, Vladivostok 690041, Russia; ^5^​ All-Russian Collection of Microorganisms (VKM), G.K. Skryabin Institute of Biochemistry and Physiology of Microorganisms, Pushchino Scientific Center for Biological Research of the Russian Academy of Sciences, Pushchino 142290, Russia; ^6^​ California Department of Food and Agriculture, Plant Pest Diagnostic Center, Sacramento, CA 95832, USA; ^7^​ Center of Parasitology of A.N. Severtsov Institute of Ecology and Evolution of the Russian Academy of Sciences, Leninskii Prospect 33, Moscow 117071, Russia

**Keywords:** intracellular micro-organism, *Betaproteobacteria*, maternal transmission, *Xiphinema*, MLSA

## Abstract

An intracellular bacterium, strain IAS^T^, was observed to infect several species of the plant-parasitic nematode genus *Xiphinema* (*Xiphinema astaregiense*, *Xiphinema incertum*, *Xiphinema madeirense*, *Xiphinema pachtaicum*, *Xiphinema parapachydermum* and *Xiphinema vallense*). The bacterium could not be recovered on axenic medium. The 16S rRNA gene sequence of IAS^T^ was found to be new, being related to the family Burkholderiaceae, class Betaproteobacteria. Fungal endosymbionts *Mycoavidus cysteinexigens* B1-EB^T^ (92.9 % sequence identity) and ‘*Candidatus* Glomeribacter gigasporarum’ BEG34 (89.8 % identity) are the closest taxa and form a separate phylogenetic clade inside Burkholderiaceae. Other genes (*atpD*, *lepA* and *recA*) also separated this species from its closest relatives using a multilocus sequence analysis approach. These genes were obtained using a partial genome of this bacterium. The localization of the bacterium (via light and fluorescence *in situ* hybridization microscopy) is in the *X. pachtaicum* females clustered around the developing oocytes, primarily found embedded inside the epithelial wall cells of the ovaries, from where they are dispersed in the intestine. Transmission electron microscopy (TEM) observations supported the presence of bacteria inside the nematode body, where they occupy ovaries and occur inside the intestinal epithelium. Ultrastructural analysis of the bacterium showed cells that appear as mostly irregular, slightly curved rods with rounded ends, 0.8–1.2 µm wide and 2.5–6.0 µm long, possessing a typical Gram-negative cell wall. The peptidoglycan layer is, however, evident only occasionally and not detectable by TEM in most cells. Another irregularly occurring shell surrounding the endosymbiont cells or the cell clusters was also revealed, probably originating from the host cell membrane. Flagella or spore-like cells do not occur and the nucleoid is diffusely distributed throughout the cell. This endosymbiont is transmitted vertically through nematode generations. These results support the proposal of IAS^T^ as a new species, although its obligate intracellular and obligate endosymbiont nature prevented isolation of a definitive type strain. Strain IAS^T^ is therefore proposed as representing ‘*Candidatus* Xiphinematincola pachtaicus’ gen. nov., sp. nov.

## Introduction

**Fig. 1.**
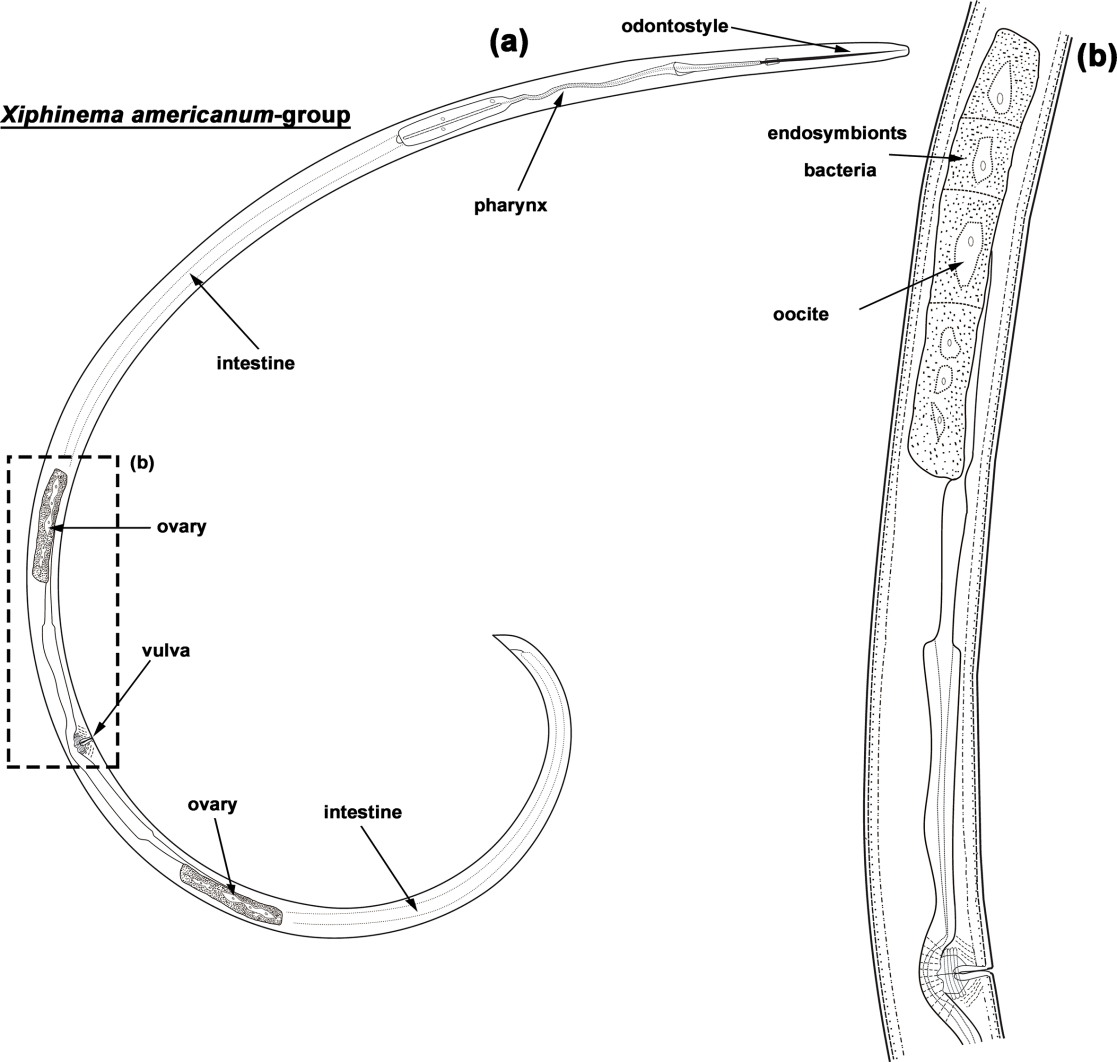
General morphology (**a**) and reproductive system (**b**) of an entire *Xiphinema americanum* group species type female (from [1]; with permission of Willey).

Bacterial endosymbionts of plant-parasitic nematodes represent a field of research that has become active in recent years [1, 2]. Several intracellular endosymbiont bacteria of different groups have been found in plant-parasitic nematodes, including: ‘*Candidatus* Cardinium hertigii’ from cyst (*Globodera* species, *Heterodera* species) and lesion (*Pratylenchus* species) nematodes; ‘*Candidatus* Wolbachia pipientis’ from lesion (*Pratylenchus* species) and burrowing (*Radopholus* species) nematodes; and ‘*Candidatus* Xiphinematobacter’ species from dagger nematodes (*Xiphinema* species) [1–8]. Recently, several publications have been devoted to ‘*Candidatus* Xiphinematobacter’ species [1, 9, 10], which are only found in representatives of the *Xiphinema americanum* group, a large species complex comprising about 60 nominal taxa [11] (Fig. 1). Several species of the *X. americanum* group are of particular interest because they are vectors of *Nepovirus* [12] and rated as A1 or A2 quarantine nematodes by the European and Mediterranean Plant Protection Organization. These nematodes reproduce by thelytokous parthenogenesis and, therefore, endosymbiont bacteria are maternally inherited [7]. In addition to three valid known species [7], the analysis of 16S rRNA gene sequences obtained from different *Xiphinema* isolates provided solid evidence for distinguishing more than 18 putatively new ‘*Candidatus* Xiphinematobacter’ species [9–11].

In addition to the aforementioned ‘*Candidatus* Xiphinematobacter’ species and related organisms, a new group of endosymbionts associated with six *Xiphinema* species: *Xiphinema astaregiense* Archidona‐Yuste, Navas‐Cortés, Cantalapiedra‐Navarrete, Palomares‐Rius and Castillo 2016, *Xiphinema incertum* Lamberti, Choleva and Agostinelli 1983, *Xiphinema madeirense* Brown, Faria, Lamberti, Halbrendt, Agostinelli and Jones 1992, *Xiphinema pachtaicum* (Tulaganov 1938) Kirjanova 1951, *Xiphinema parapachydermum* Gutiérrez-Gutiérrez, Cantalapiedra-Navarrete, Decraemer, Vovlas, Prior, Palomares-Rius and Castillo 2012 and *Xiphinema vallense* Archidona‐Yuste, Navas‐Cortés, Cantalapiedra‐Navarrete, Palomares‐Rius and Castillo 2016 from a separate phylogenetic lineage of the *X. americanum-*group species was recently revealed and characterized by molecular methods [1]. These endosymbionts belong to the class *Betaproteobacteria*, family Burkholderiaceae, in contrast to ‘*Candidatus* Xiphinematobacter’ species affiliated with the class Verrucomicrobia. Preliminary phylogenetic analysis based on partial 16S rRNA gene sequences suggests that these new endosymbionts represent a rare novel species within the family Burkholderiaceae [1].

In this work we present a detailed characterization of the endosymbiont bacterium found in the nematode *X. pachtaicum* from the rhizosphere of sour orange trees (*Citrus* × *aurantium* L.) from Cordoba, Spain, and, based on morphological, phylogenetic and genomic characteristics propose a novel candidate genus and species for this uncultured bacterium (strain IAS^T^).

## Methods

### Nematode population sampling, extraction and morphological identification

Specimens of the nematode host, *X. pachtaicum*, were isolated from infested soil samples collected at the experimental farm of the Institute for Sustainable Agriculture-CSIC (Córdoba, Spain) from the rhizosphere of sour orange trees. Nematodes were extracted from soil by a modification of Cobb’s decanting and sieving method [13].

Specimens were examined using an Olympus BX50 light microscope with differential interference contrast at magnifications up to ×1000. Photographs were taken with an Olympus DP70 camera and Cell software (Olympus Software Imaging for Life Sciences). All measurements were expressed in micrometres (µm). Specimens were individually selected and kept alive for transmission electron microscopy (TEM) analysis and frozen at −20 °C for molecular analysis. For preliminary genome sequencing a bulk of nematodes (more than 5000 individuals) was hand-picked, concentrated in a small volume of PCR-grade water and kept at −20 °C. We use this sampling approach because these long-lived nematodes cannot be easily grown as single-maternal lineages.

### Culturing methods for *X. pachtaicum* bacterial endosymbionts

Two conventional enriched culture media were recently tested by Palomares-Rius *et al*. [1] to attempt the multiplication of this bacteria outside of its nematode host: Luria–Bertani (LB) medium (1 % peptone, 0.5 % yeast extract, 0.5 % NaCl and 1.2 % Bacto agar) and yeast–glucose–peptone medium (0.5 % yeast extract, 0.4 % glucose, 0.1 % NaCl and 1.5 % Bacto agar). This article follows similar protocols to Palomares-Rius *et al*. [1] and Vicente *et al*. [14] using sodium hypochlorite (0.5 % v/v) or 3 % hydrogen peroxide as disinfectants, respectively; in both protocols the nematodes were surface-sterilized by soaking them for 5 min in the disinfectant, followed by washing five times with sterile distilled water. An accurate disinfection method ensures effective nematode surface coat sterilization and then avoids the isolation of bacteria from the nematode surface. After rinsing, nematodes were cut using a sterile blade on sterile glass slides under the microscope using aseptic conditions and then were plated. For testing the nematode, surface sterilization methods were included as controls by plating individual nematodes without dissecting in each culture media tested. The testing media were LB, tryptic soy agar (1.5 % tryptone, 0.5 % NaCl, 0.5 % soytone and 1.5 % agar), Reasoner’s 2A agar (1.5 % agar, 0.05 % casein acid hydrolysate, 0.05 % dextrose, 0.03 % K_2_HPO_4_, 0.0024 % MgSO_4_ anhydrous, 0.05 % proteose peptone, 0.03 % sodium pyruvate, 0.05 % soluble starch, 0.05 % yeast extract), yeast–glucose–peptone (0.5 % yeast extract, 0.4 % glucose, 0.1 % NaCl and 1.5 % Bacto agar), charcoal–yeast extract agar (1 % yeast extract, 0.2 % charcoal activated, 1.5 % agar) and buffered charcoal–yeast extract agar with l-cysteine (1 % yeast extract, 0.2 % charcoal activated, 1 % ACES buffer, 0.1 % α-ketoglutarate monopotassium salt, 0.025 % Fe_4_(P2O7)_3_, 1.5 % agar, 0.04 % l-cysteine hydrochloride). Plates were then kept at 27 °C and checked daily during 12 days for bacterial growth. All procedures were conducted in a sterile environment.

### TEM

Adult females and juveniles of *X. pachtaicum* were fixed in аn ice-cold Karnovsky fixative (2.5 % glutaraldehyde and 2 % paraformaldehyde in 0.05 M sodium cacodylate buffer (pH 7.4) with 0.25 mg ml^−1^ MgCl_2_). After 30 min of pre-fixation by the stock fixer diluted 1 : 1 with 0.05 M sodium cacodylate buffer, the head and tail of each animal were removed, the mid-parts being cut into halves at the vulva level. Thereafter, the pieces–each containing one branch of the female genital system–were fixed in a fresh portion of full Karnovsky fixative overnight at 4 °C. After rinsing in sodium cacodylate buffer solution and post-fixation in 1 % osmium tetroxide in the same buffer for 1 h at room temperature, the specimens were stained *en bloc* for 1 h in 1 % aqueous uranyl acetate. Then specimens were dehydrated in increasing concentrations of ethanol followed by isopropanol series and embedded in Spurr resin. Thin longitudinal sections cut with a diamond knife (Diatome) using a Leica UC6 ultramicrotome were stained with uranyl acetate and lead citrate and examined by TEM (JEM 1010, JEOL) equipped with a charge-coupled device side-mounted Veleta camera (EMSIS). Parts of the specimens were observed with a Zeiss Sigma 300 VP electron microscope.

### Fluorescence *in situ* hybridization (FISH) and confocal laser microscopy

FISH was performed using the high-stringency protocols of Vandekerckhove *et al*. [7] and Brown *et al*. [15]. Mixed stages, including juveniles and gravid females of *X. pachtaicum* (IAS), were surface sterilized in 1 ml 0.1 % w/v benzalkonium chloride for 1 min, washed twice with 1 ml of 0.85 % w/v NaCl for 2 min, permeabilized by fixation in 1 ml of 1 : 1 v/v glacial acetic acid and washed twice in 1 ml of 100 % ethanol for 5 min each. Samples were then treated with a 10 min soak in 1 ml of 1 : 1 v/v 100 % methanol and phosphate-buffered saline solution with Tween 20 (PBT; 150 mM NaCl, 10 mM Na_2_HPO_4_, 0.1 % w/v Tween 20, with HCl to adjust to pH 7.4). Next, specimens were fixed in 1 ml of 1 % v/v formaldehyde and PBT for 30 min, followed by two washes in 1 ml PBT for 2 min. Hybridization was performed for 3 h at 42 °C using 0.2 ml prewarmed hybridization buffer [20 mM Tris-HCl pH 7.4, 0.02 % w/v sodium dodecyl sulphate (SDS), 0.9 M NaCl, 5 mM ethylenediaminetetraacetic acid (EDTA), 60 % v/v formamide] and 0.02 ml of the probe at 10 mM in TE (10 mM Tris-HCl, 1 mM EDTA, pH 7.4). The FISH probe Burkho_1 5′-5ATTO550/RCCCTCTGTACCGACCAT-3′ was designed by Palomares-Rius *et al*., [1] to target the 16S rRNA of the Burkholderiaceae endosymbiont (in red) in *Xiphinema* nematodes. After hybridization, specimens were washed twice at 46 °C for 30 min in 1 ml pre-warmed hybridization wash buffer (20 mM Tris-HCl, 0.02 % w/v SDS, 0.008 M NaCl, 5 mM EDTA). Finally, specimens were mounted on slides in Vectashield (Vector Laboratories) with DAPI and were analysed from three-dimensional confocal optical stacks collected using an Axioskop 2 MOT microscope (Carl Zeiss) equipped with an argon laser and controlled by Carl Zeiss Laser Scanning System LSM5 Pascal software. Confocal laserscanning microscopy data were recorded and transferred for analysis to Zeiss LSM Image Browser version 4.0 (Carl Zeiss). Figures were processed with Photoshop 22.4.1 software (Adobe Systems).

### DNA purification and partial genome assembly

DNA extraction was performed using liquid N_2_ for grinding the nematodes in a mortar and pestle until a fine powder was obtained. Then, DNA was isolated using DNaesy blood kit (Qiagen) following the manufacturer’s instructions. After quality checking and quantification, fragment library construction using Truseq DNA Library Kit (Illumina) was done following the manufacturer’s instructions. This shotgun paired-end library (2×300 bp) was sequenced on an Illumina MiSeq instrument at the STAB-Vida sequencing facilities (C. Caparica, Portugal). The total number of sequences read was 28 796 568. Sequences were quality-trimmed and -filtered as follows [min. length: 150 bp; remove leading low quality or N bases (below quality 3): 10; remove trailing low quality or N bases (below quality 3): 10; scan the read with a four-base-wide sliding window; cutting when the average quality per base drops below 20] using Trimmomatic version 0.35 [16]. MIRA assembler version 4.9.6 [17] was the best among several preliminary tests (Velvet, SOAPdenovo; results not shown). The statistics of this assembly were as follows: N50, 793 bp; maximum contig length, 128 454 bp; contig count of 189 711; total contig length, 107 921 826 bp. The partial bacterial genome was selected from these contigs using a BLASTX search with one of the closest 16S rRNA relative with a full genome available (‘*Candidatus* Glomeribacter gigasporarum’, accession GCA_001684025.1). This strategy gave us the partial genome of the endosymbiont with the following statistics: G+C content, 39.4 mol%; length N50, 56595 bp; maximum length, 128 454 bp; length mean, 10 689 bp; length median, 1987 bp; minimum length, 112 bp; number of bp, 769656; number of sequences, 72). From this partial genome, five housekeeping genes [ATP synthase subunit beta (*atpD*), gyrase B (*gyrB*), GTP binding protein (*lepA*), recombinase A (*recA*) and RNA polymerase β subunit (*rpoB*)] and the 16S rRNA gene sequence were selected for multilocus sequence analysis (MLSA) as described previously [18, 19] with the close phylogenetic related species of this endosymbiont (*Mycoavidus cysteinexigens* and *Mycetohabitans rhizoxinica*) [20]. However, only three genes were found in this preliminary and incomplete endosymbiont genome (*atpD*, *lepA* and *recA*; MW485035–MW485037) and the 16S rRNA gene was already obtained by Palomares-Rius *et al*. [1] (KT735068 and KT735072). The two remaining genes (*gyrB* and *rpoB*) were tentatively amplified by PCR following Estrada-De-Los Santos *et al*. [19], but the results were unsuccessful (data not shown). All bioinformatics analysis was done using the supercomputing platform at the Servicio de Supercomputación y Bioinformática-University of Malaga.

### Phylogenetic analyses

The partial 16S rRNA gene was amplified using 8F and 1492R primers [21, 22] as described by Palomares-Rius *et al*. [1]. The initial denaturation step was at 94 °C for 4 min, followed by 35 cycles of 94 °C for 30 s, 50 °C for 30 s and 68 °C for 45 s, and an elongation step at 68 °C for 8 min. The sequences of this gene were obtained for *X. pachtaicum* IAS-CSIC from Palomares-Rius *et al*. [1] and deposited in GenBank [1, 10]. The other three genes (*atpD*, *lepA* and *recA*) were obtained from the partially assembled genome of the *X. pachtaicum* population IAS-CSIC as described previously. Percentage similarity between sequences was calculated using the sequence identity matrix in BioEdit. For that, the score for each pair of sequences was compared directly and all gap or place-holding characters were treated as a gap. When the same position for both sequences had a gap, it was not treated as a difference. Outgroup taxa for each dataset (16S rRNA and concatenated genes) were chosen following previous published studies [1, 19]. In the case of the concatenated alignment (16S rRNA, *atpD*, *lepA* and *recA*) sequence alignments of the different genes were made using the FFT-NS-2 algorithm of mafft version 7.450 [23] and the selected sequences are shown in Table S1 (available in the online version of this article). Sequence alignments were visualized using BioEdit [24] and edited by Gblocks version 0.91b [25] in the Castresana Laboratory server (http://molevol.cmima.csic.es/castresana/Gblocks_server.html) using options for a less stringent selection (minimum number of sequences for a conserved or a flanking position, 50 % of the number of sequences+1; maximum number of contiguous non-conserved positions, 8; minimum length of a block, 5; allowed gap positions, with half). All genes were concatenated using SequenceMatrix [26]. Phylogenetic analyses of the sequence datasets were performed based on the maximum-likelihood (ML) method using PAUP* 4b10 [27] and Bayesian inference (BI) using MrBayes 3.1.2 [28]. The best-fit model of DNA evolution was obtained using JModelTest version 2.1.7 [29] with the Akaike information criterion (AIC). The Akaike-supported model, the base frequency, the proportion of invariable sites and the gamma distribution shape parameters and substitution rates in the AIC were then used in the phylogenetic analyses. BI analysis under a general time-reversible with invariable sites and a gamma-shaped distribution (GTR+I+G) model for the 16S rRNA and *atpD*, *recA* and *lepA* datasets were run with four chains for 2×10^6^ generations, respectively. The Markov chains were sampled at intervals of 100 generations. Two runs were conducted for each analysis. After discarding burn-in samples and evaluating convergence, the remaining samples were retained for further analyses. The topologies were used to generate a 50 % majority-rule consensus tree. Posterior probabilities are given on appropriate clades. In the ML analysis, the estimation of the support for each node was obtained by bootstrap analysis with 100 fast-step replicates. Trees from all analyses were visualized using FigTree software version 1.42 (http://tree.bio.ed.ac.uk/software/figtree/).

## Results

### Culturing methods for *X. pachtaicum* bacterial endosymbionts

The capacity of the bacterial endosymbionts from *X. pachtaicum* to multiply outside of their host tissues was investigated. Several media suitable to sustain growth of a wide spectrum of different micro-organisms, including Gram-negative bacteria belonging to phylum Proteobacteria (e.g. *Legionella* species, rhizobia, *Burkholderia* species), additionally supplemented in some case for their selective isolation and growth, were tested. *Xiphinema pachtaicum* bacterial endosymbiont growth in the culture media was checked daily during 12 days but, unfortunately, growth was never observed in any of the tested media under the chosen specific conditions.

### DAPI fluorescence microscopy

**Fig. 2.**
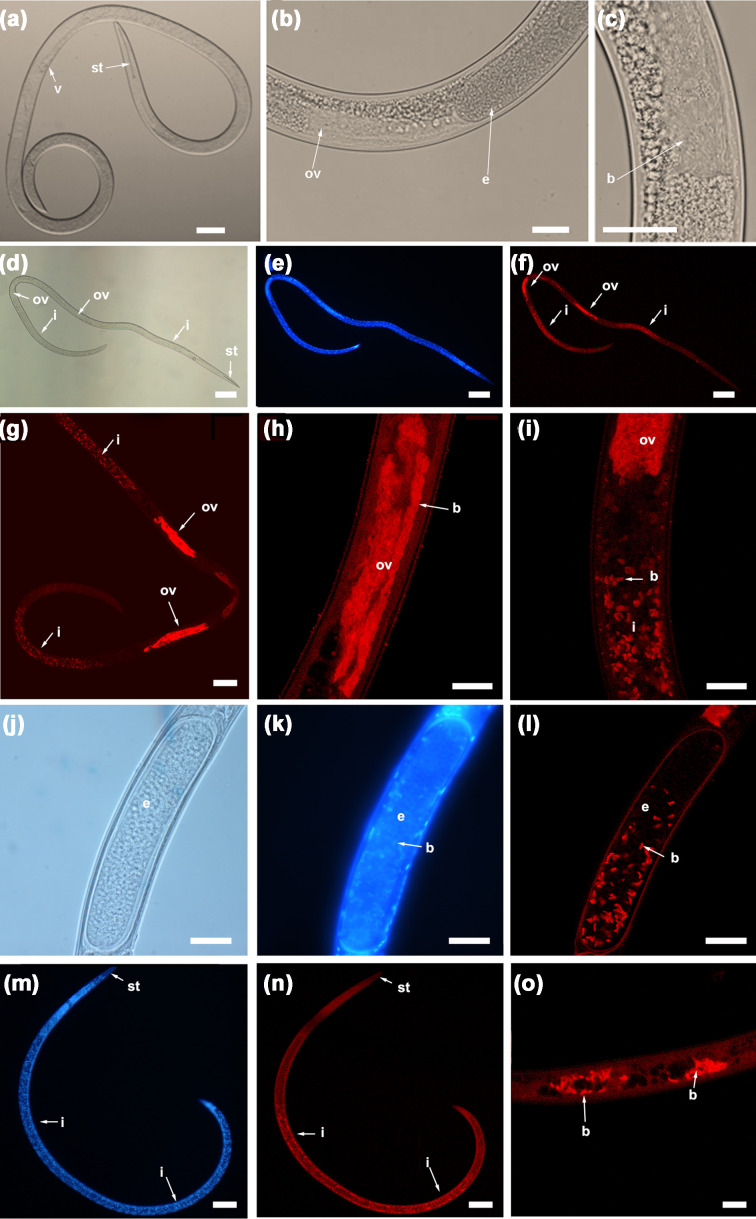
Detection of bacterial endosymbionts of *Xiphinema pachtaicum* (‘*Candidatus* Xiphinematincola pachtaicus’ gen. nov., sp. nov.) in FISH using the Burkho_1 probe and analysed by confocal laser scanning microscopy. (**a**) Female showing the typical characteristics of the *X. americanum* group. The vulva and the odontostyle are arrowed. (**b**) Light microscopy picture showing in detail the region of the nematode body including the ovary and a developing egg (arrowed). (**c**) Detailed light microscopy picture showing bacterial endosymbionts inside the ovary (arrowed). (d–f) Light, epi‐fluorescent DAPI stained (blue) and confocal FISH results (red) showing the presence of bacterial endosymbionts in the ovaries and the intestine (both arrowed), which occupies mostly the whole female nematode body. (**g**) A detailed confocal FISH picture of a *X. pachtaicum* female showing bacterial colonization (red) in the ovaries and the intestine (both arrowed). (**h**) Confocal FISH detailed picture of an ovary with endosymbionts (arrowed). (**i**) Detailed confocal FISH picture of an ovary with endosymbionts (red) and the proximal nematode intestinal area, which has a lower density of endosymbiont (red) than in the rest of the intestine. (j–l) Light, epi‐fluorescent DAPI stained (blue) and confocal FISH results (red) showing the presence of bacterial endosymbionts (arrowed) in an early developing embryo. (m‐n) Epi‐fluorescent DAPI stained (blue) and confocal FISH results (red) in a juvenile specimen showing the presence of bacterial endosymbionts along the whole intestine (arrowed). (**o**) Detailed confocal FISH picture of a section of a juvenile intestine with endosymbionts (arrowed). b, bacterial endosymbionts; e, Egg; i, intestine; ov, ovary; st, odontostyle; v, vulva. Scale bars: a, d–g, m, n, 50 µm; b, c, h–l, 20 µm; o, 30 µm (from [1], with permission of Wiley).

FISH results (Fig. 2) showed that the FISH probe Burkho_1 was specific to the Burkholderiaceae-related endosymbionts associated with *X. pachtaicum* and was localized specifically to bacterial cells within the nematode gut lumen, ovarian sheath, eggs and developing embryos. The DAPI staining co-localized consistently with the FISH probe. Bacterial cells in the ovaries were highly packed in comparison with the cells in the gut lumen. The density of bacteria in the gut lumen decreased, closer to the ovaries (Fig. 2). Additionally, pre-rectum sections showed that there was a lower density of bacteria in adults and juveniles (Fig. 2). The natural autofluorescence of some nematode organs does not interfere with the positive signals obtained in the FISH experiments presented in this work and can be used as a tool to help in the localization of internal structures within the nematode body without any additional staining.

### Electron microscopy

**Fig. 3.**
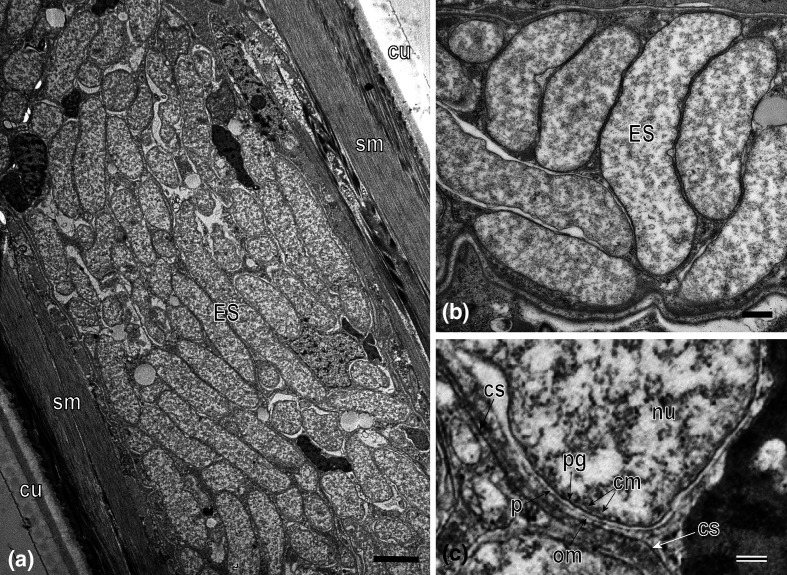
Microscopic TEM observations of ‘*Candidatus* Xiphinematincola pachtaicus’ gen. nov., sp. nov. in *Xiphinema pachtaicum*. (**a**) Overview of the ovary. (**b**) Cluster of endosymbiont cells in the ovary. (**c**) Detailed endosymbiont cell membranes. cm, Electron dense cytoplasm; cs, circumjacent shell surrounding the endosymbiont cells and cell clusters (rather derived from the host cell membrane); cu, cuticle; ES, endosymbiont cells; nu, nucleoid; om, outer membranes; p, extensive periplasm; pg, peptidoglycan; sm, somatic muscles. Scale bars: a, 2 µm; b, 0.5 µm; c, 200 nm.

TEM observations supported the presence of bacteria inside the nematode body where they occupy ovaries and occur inside the intestinal epithelium (Figs 3 and S1). The endosymbionts fill the ovaries of adult females almost completely as a mass of tightly packed cells (Fig. 3a, b). The pre-vitellogenic oocytes surrounded by bacteria have a nucleus with nucleolus and cytoplasm containing ribosomes with mitochondria and cisterns of rough endoplasmic reticulum (Fig. S1a, b). At a lower density, the bacteria occurred inside the intestinal epithelial cells as individual cells or cell clusters (Fig. S1c, d). The females studied by TEM contained no bacterial cells in tissues other than in the ovaries and intestine.

In the nematode tissues, the endosymbiont cells appear mostly as irregular, slightly curved rods with rounded ends, 0.8–1.2 µm wide and 2.5–6.0 µm long (Figs 3a, b and S1a, b). Smaller spherical or coccobacillary forms of varying size also occurred as a result of binary or uneven binary divisions of mother cells. Longer filiform cells of up to 9.0 µm were occasionally present due to the delayed cell division.

The cell-wall architecture of the *X. pachtaicum* endosymbiont is typical of Gram-negatives (Fig. 3c). It includes the electron‐dense cytoplasmic and outer membranes, the extensive periplasm, and a thin peptidoglycan layer adjacent to the cytoplasmic membrane. The peptidoglycan layer is, however, evident only occasionally and not detectable by TEM in most cells. Another irregularly-occurring shell (cs) surrounding the endosymbiont cells or the cell clusters was also revealed. It resembles a vacuole membrane detected in the thin sections of *Xiphinema* populated with the '*Candidatus* Xiphinematincola' endosymbionts, which supposedly derives from the host cell membrane [6]. Flagella or spore-like cells do not occur. The nucleoid is diffusely distributed throughout the cell. No other intracytoplasmic inclusions have been observed.

### Molecular differences and 16S rRNA-concatenated, 16S rRNA, *atpD*, *lepA* and *recA* phylogenetic analysis

**Fig. 4.**
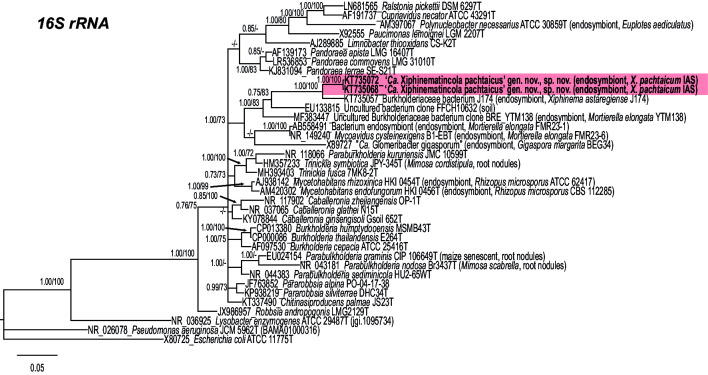
Phylogenetic relationships of ‘*Candidatus* Xiphinematincola pachtaicus’ gen. nov., sp. nov. and related bacterial species. Bayesian 50 % majority rule consensus trees as inferred from 16S rRNA sequences alignments under the GTR+I+G model. Posterior probabilities (BI) and bootstrap values (ML) more than 50 % are given for appropriate clades.

**Fig. 5.**
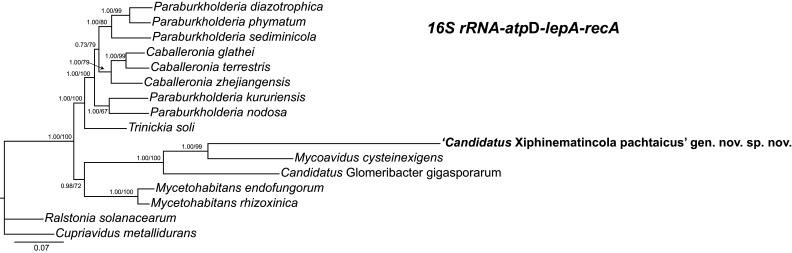
Phylogenetic relationships of ‘*Candidatus* Xiphinematincola pachtaicus’ gen. nov., sp. nov. and related bacterial species. Bayesian 50 % majority rule consensus trees as inferred from 16S rRNA*–atpD–recA–lepA* sequences alignments under the GTR+I+G model. Posterior probabilities more than 70 % are given for appropriate clades and bootstrap values for maximum-likelihood phylogeny more than 70 % are given for appropriate clades. Newly obtained sequences in this study are in bold.

The 16S rRNA-based phylogenetic analysis demonstrated that strain IAS^T^ from *X. pachtaicum* together with fungal endosymbionts *M. cysteinexigens* B1-EB^T^ (92.9 % sequence similarity) and ‘*Candidatus* Glomeribacter gigasporarum’ BEG34 (89.8 % similarity), formed a separate phylogenetic branсh within the radiation of genera of the family *Burkholderiaceae*, class *Betaproteobacteria* (https://lpsn.dsmz.de/family/burkholderiaceae) (Figs 4 and 5). This phylogenetic branch also includes some other uncultured *Burkholderiaceae* or associated with fungi and nematodes. IAS^T^ was closest to other endosymbiont strains associated with different populations of *X. pachtaicum* and five other nematode species (*X. astaregiense*, *X. incertum*, *X. madeirense*, *X. parapachydermum* and *X. vallense*) [1], only included the relatively distant Burkholderiaceae bacterium J174 from (96.3 % similarity) in this phylogenetic analysis.

Among the other *Burkholderiaceae* species with validly published names (clustering separately from the aforementioned phylogenetic branch and having high, more than 60 mol% G+C DNA content), IAS^T^ showed the closest relatedness to the endohyphal bacteria *M. rhizoxinica* HKI 454^T^ (92.8 % similarity) and *Mycetohabitans endofungorum* HKI 456^T^ (92.3 % similarity) from the fungus *Rhizopus microsporus*. The similarities of IAS^T^ to other validly described species did not exceed 92.0 % (found against *Caballeronia ginsengisoli* Gsoil 652^T^), with 91.0 % determined to *Burkholderia cepacia* ATCC 25416^T^ (AF097530; the type strain of type species of type genus of Burkholderiaceae). All the above similarity values are well below the estimated threshold levels (94.5–95.0 %) for distinct prokaryote genera [30, 31]. These values are below the similarities between *B. cepacia* ATCC 25416^T^ and type species of some Burkholderiaceae genera established in the last decade *de novo* or as results of reclassification of *Burkholderia sensu lato* (e.g. *Paraburkholderia* [32], *Caballeronia* [33, 34], *Robbsia* [35], *Pararobbsia* [36] and *Chitinasiproducens* [37]). The distinctness between type strains of species of the above genera [showing high 16S rRNA sequence similarity, from ~96 to 97.5 % (between *B. cepacia* ATCC 25416^T^ and *Trinickia fusca* 7MK8-2^T^)] was strongly supported by genome analysis [34, 36–39].

Thus, the above data clearly indicate that the bacterial endosymbiont IAS^T^ is different from all other genera with validly published names and *Canditatus* taxa of the family *Burkholderiaceae* and represents a new genus and a new species in this family. As follows from the data reported previously [1], this new *Candidatus* genus also comprises a few other putative species (which so far have not been scientifically named) associated with some nematode species of the *Xiphinema americanum* group. '*Candidatus* Xiphinematincola pachtaicus' gen. nov., sp. nov. can be recognized among these unnamed putative species (at least) by comparison with the concatenated set of sequences of housekeeping genes 16S rRNA, *atpD*, *lepA* and *recA* in a highly supported clade with *M. cysteinexigens* and these two species with *Candidatus* Glomeribacter gigasporarum (Fig. 5). These three species are related to *M. rhizoxinica* and *M. endofungorum*, species also found in fungi and differing in the phylogenetic position from the 16S rRNA tree.

Contigs obtained from our preliminary assembly (more sequencing is ongoing) are only a small part of the putative genome of ‘*Candidatus* Xiphinematincola pachtaicus’ gen. nov., sp. nov. This strategy gave us the partial genome of the endosymbiont (G+C content, 39.4 mol%; length N50, 56 595 bp; maximum length, 128454; length mean, 10689; length median, 1987; minimum length, 112 bp; number of bp, 769 656 bp; number of sequences, 72).

## Discussion

Results from our integrative approaches (FISH, TEM, molecular and ecological properties) support the proposal that strain IAS^T^ constitutes a new bacterial species representing a new genus within the family *Burkholderiaceae* as a specific endosymbiont in nematode species of the *X. americanum* group. The various media tested in this article, alongside results of previous other studies [1], confirm this species as a putative strict endosymbiont with vertical transmission. However, no other metabolic tests could be assessed because of the difficulty in isolating and cultivating this bacterium from inside the nematode ovaries, where the bacteria localize at the maximum density.

In *X. pachtaicum* females, the bacteria cluster around the developing oocytes and were primarily found embedded inside the epithelial wall cells of the ovaries, while they are dispersed in the intestine. Using light microscopy (LM) alone, these bacteria are only detected in some specimens of *X. pachtaicum*, as the dark nature of the intestinal contents render their detection difficult. To overcome this problem, results from FISH probes, co-localized with DAPI and LM images, demonstrated the correct localization of the bacteria inside the body of the nematode. The density of bacteria in the gut lumen decreased closer to the ovaries, but no active movement was found when the nematode ovary was cut and the bacteria were expelled outside the nematode body. TEM confirmed the location of bacteria within the host nematode body, where they preferably inhabit the ovaries of adult females. The cell-wall architecture of the *X. pachtaicum* endosymbiont is typical of Gram-negative bacteria, which is consistent with its phylogenetic position within the family *Burkholderiaceae*. The reduced or even absent peptidoglycan layer was reported in some endosymbionts, such as '*Candidatus* Xiphinematobacter' [6]. This feature is also characteristic of obligate intracellular pathogens replicating within the interior of living cells, an osmotically protected niche [40]. The lack of peptidoglycan, a potent stimulator of the eukaryotic immune system, is also essential for survival and growth of bacteria in the host cells without a risk of detection and destruction by the host’s protective systems [40]. In addition, the irregularly observed shell surrounding cell clusters is very similar to that revealed around the cell clusters of the endosymbiont '*Candidatus* Xiphinematobacter' within the *Xiphinema* body [6]. Thus, the reduced (or absent) peptidoglycan layer and the host-derived shell surrounding the bacterial cells may serve as other features indicative of an endosymbiotic lifestyle for the target bacterium.

The phylogenetic analysis placed the new taxon close to other endosymbionts species of fungi and with members of the *Candidatus* phyla (*M. cisteinexigens*, ‘*Candidatus* Glomeribacter gigasporarum’, *M. rizhoxinica* and *M. endofungorum*). The relationship of ‘*Candidatus* Xiphinematincola pachtaicus’ sp. nov. with its host has been demonstrated as a co-evolution type [1] and this bacterium has a clearly similar ecological niche as ‘*Candidatus* Xiphinematincola’ species, which colonizes closely related host species of *Xiphinema* within the *X. americanum* group. These ecological features are the localization in the intestine and ovaries and the vertical transmission of the endosymbiont through eggs. ‘*Candidatus* Xiphinematobacter americani’ has been fully sequenced and showed a mutualistic relationship that provision the host with required nutrients [15]. The closest taxon found using blastn in the 16S rRNA has been an uncultured soil bacterium (EU133815). This bacterium sequence has been found from an undisturbed tall grass prairie soil in central Oklahoma, and no more data is available [41]. In this sense, the family *Burkholderiaceae* has members with a wide range of ecological features such as opportunistic human pathogens, some environmental species, plant endophytes and involved in legume nodulation or plant-pathogens [19] and the genus has been revised recently using phylogenomics [38]. The closest phylogenetically taxa to ‘*Candidatus* Xiphinematincola pachtaicus’ sp. nov. in the concatenated set of genes identified species such as *M. cysteixigens*, followed by ‘*Candidatus* Glomeribacter gigasporarum’ (Fig. 5). Both species have different ecologies, on one side, *M. cysteinexigens* (B1-EB^T^) is the endohyphal symbiont of the non-pathogenic fungus *Mortierella elongata* [42], while ‘*Candidatus* Glomeribacter gigasporarum’ lives in the cytoplasm of dormant or germinating spores and symbiotic mycelia of the fungal species *Gigaspora margarita*, *G. decipiens*, *Scutellospora persica* and *S. castanea* [43]. *Mycoavidus cysteinexigens* seems to have a parasitism role with their host reducing mycelial growth and fatty acid accumulation, but some secondary metabolites could be useful for the host [44], while ‘*Candidatus* Glomeribacter gigasporarum’ helps in the germ tube extension and increase fatty acids availability [45]. *Mycetohabitans rhizoxinica* and *M. endofungorum* are phylogenetically related using the concatenated set of genes, and they have the same host, *R. microsporus. M. rhizoxinica* (HKI 454^T^) produces rhizoxin (virulence factor, antimitotic polyketide) for *R. microsporus*, which causes blight symptoms in rice seedlings [46, 47] and controls the vegetative reproduction of the host [48]. *Mycetohabitans rhizoxinica* HKI 454 has been pathogenic to *C. elegans*, probably because of the production of rhizoxin [38]. Only some of these fungal endosymbiont genomes have been studied [20, 38, 44, 49, 50]. These Mucoromycota endosymbiotic bacteria relationships are supposed to be ancient [44, 51–53]. For example, *M. elongata* and *M. cysteinexigens* symbiosis has been dated over 350 million years ago and concomitant with the terrestrialization and diversification of land fungi and plants [44]. Interestingly, this old relationship is related to a low rate of molecular evolution in Burkholderia endosymbionts [49].

In our case, this endosymbiont–host relationship has been estimated as having existed in ‘*Candidatus* Xiphinematobacter’ species for roughly 150 million years, with no evidence of horizontal transmission or survival in the external environment [6]. In the case of ‘*Candidatus* Xiphinematincola pachtaicus’ sp. nov., a high mutation rate is detected from the close related taxa in the 16S rRNA and the concatenated set of genes, shown by the long tree branches. This data reinforces the notion that ‘*Candidatus* Xiphinematincola pachtaicus’ sp. nov. represents a strict endosymbiont with a high mutation rate and small effective population size. In our case, arbuscular mycorrhizal fungi could cover and penetrate the root cells, places where *Xiphinema* species could then feed. However, the odontostyle lumen of these nematodes is not wide enough to pass bacteria inside the digestive tract. Another possibility is the colonization of endosymbionts via other apertures in the nematode body, such as the anus or vulva. In any case, the feeding of *X. americanum*-group species does not promote the production of tip galls and specific feeding cells, unlike with other non-*X. americanum*-group species [1]. This structure would produce a richer nutritional tissue for the nematode, although species from the *X. americanum*-group appear to feed on plant roots without the need for cellular differentiation. The presence of the endosymbiont may indeed be helping to increase the nutritional value of the nematode food source in this sense. Brown *et al*.[15] suggested the phloem feeding of these nematodes and the synthesis role played by essential amino acids from the endosymbiont ‘*Candidatus* Xiphinematobacter’ species. However, histological studies are lacking to test the feeding on phloem hypothesis for these nematodes, and furthermore, their stylet structure can hardly allow them to feed in this tissue. In *M. cysteinexigens* (AG77), biosynthetic pathways have been revealed that allow their production of numerous amino acids, including histidine, cysteine, tyrosine, arginine, lysine and asparagine [44]. Such biosynthesis could be maintained and in fact demonstrate a symbiosis with the host in the case of ‘*Candidatus* Xiphinematincola pachtaicus’ sp. nov. This relationship could be investigated further, and demonstrated in a full genome sequencing of the endosymbiont and the host nematode.

## Description of ‘*Candidatus* Xiphinematincola’ gen. nov.

*Xiphinematincola* (Xi.phi.ne.mat.in'co.la. N.L. neut. n. *Xiphinema* a nematode genus; L. masc. n. *incola* dweller, inhabitant; N.L. masc. n. Xiphinematincola the one who lives in nematode of the genus *Xiphinema*).

Rod-shaped, Gram-negative. Occurs as obligate endosymbionts of the nematode *X. pachtaicum* (Nematoda, Longidoridae) and a few other species of the *X. americanum* group, as follows from the 16S rRNA gene sequence analysis [1]. Transmitted vertically through nematode generations. The DNA G+C content (39.4 % as determined for the type species) is lower than that in the recognized and *Canditatus* taxa of Burkholderiaceae.

Based on the 16S rRNA gene sequence analysis, the genus belongs to the family Burkholderiaceae*,* order *Burkholderiales*, class *Betaproteobacteria* and is clearly different from all other genera with validly published names and *Canditatus* taxa composing Burkholderiaceae.

The type species is '*Candidatus* Xiphinematincola pachtaicus’

## Description of ‘*Candidatus* Xiphinematincola pachtaicus’ sp. nov.

*Xiphinematincola pachtaicus* (N.L. masc. adj. pachtaicus based on the specific epithet of *Xiphinema pachtaicum*, originally described as *Longidorus pachtaicus*).

Non-sporulating, straight or slightly curved rods with rounded ends, 0.8–1.2 µm wide and 2.5–6.0 µm long measured in the nematode tissue (TEM). The bacteria multiply by binary or uneven binary division, producing spherical and coccobacillary forms varying in size. Longer cells, up to 9.0 µm, may occur due to the delayed cell division. The cell-wall structure is typical of Gram-negative bacteria. The cell wall includes the cytoplasmic and outer membranes, extensive periplasm, and a peptidoglycan layer adjacent to the cytoplasmic membrane. The peptidoglycan layer is usually reduced and observable only in some cells. Clusters of the bacterial cells in the nematode tissues are surrounded by a shell originating from the host cell membrane.

Preferably inhabits ovaries of adult female, clustering around the developing oocytes and locating inside the epithelial wall cells of the ovaries and with lower density occur in intestinal epithelial cells. Transmitted vertically through nematode generations.

Found in *X. pachtaicum* collected in the rhizosphere of sour orange trees, Avenida Menendez Pidal, Córdoba, Spain (37.860029, –4.796813). Can be recognized among other existing putative species of this genus [1] by comparison of sequences of 16S rRNA and the concatenated set of housekeeping genes 16S rRNA, *atpD*, *lepA* and *recA* (GenBank sequence numbers) and their unique ecology.

## Supplementary Data

Supplementary material 1
