# Supplementary material for: ‘Candidatus Xiphinematincola pachtaicus' gen. nov., sp. nov., an endosymbiotic bacterium associated with nematode species of the genus Xiphinema (Nematoda, Longidoridae)
Source: Int J Syst Evol Microbiol. 2021 Jul 21;71(7):004888. doi: 10.1099/ijsem.0.004888 (PMC8489844; doi:10.1099/ijsem.0.004888)
Supplement: Supplementary material 1 [file ijsem-71-4888-s001.pdf]

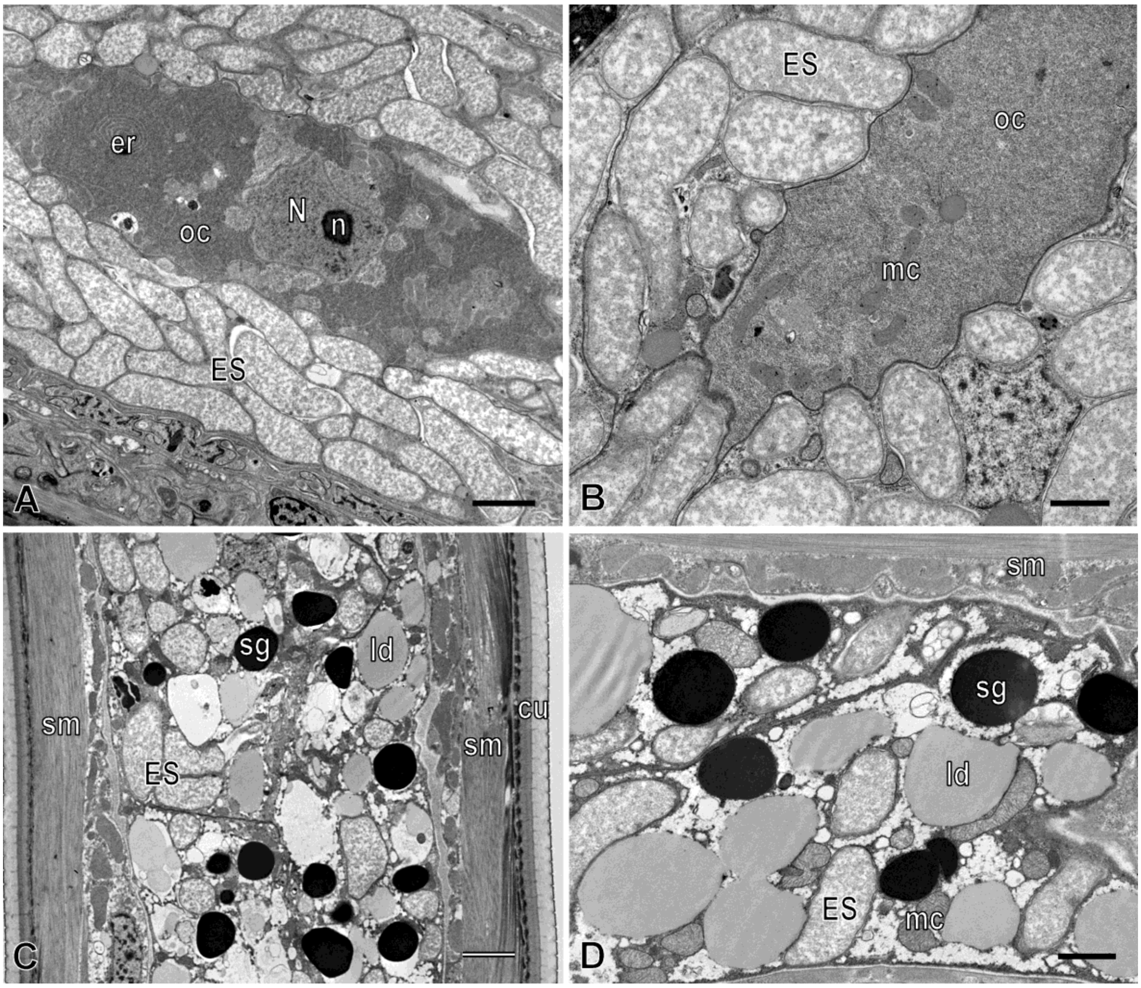

Fig. S1. Microscopic TEM observations of '*Candidatus* Xiphinematincola pachtaicus' gen. nov., sp. nov. in *Xiphinema pachtaicum*. (A and B) Ovary; (C and D) Intestine. (A) Pre-vitellogenic oocyte surrounded by bacterial endosymbiont cells, overview; (B) Endosymbiont cells closely packed around the pre-vitellogenic oocyte, higher magnification; (C) Intestinal epithelium with occasional intracellular endosymbiont cells, overview; (D) Endosymbiont cells inside the intestinal cells, higher magnification. cu, cuticle; er, rough endoplasmic reticulum; ES, endosymbionts; ld, lipid droplets; mc, mitochondria; N, nucleus of oocyte; n, nucleolus; oc, cytoplasm of oocyte; sg, storage granules; sm, somatic muscles. Scale bars: A, C = 2 μm; B, D = 1 μm.

Table S1. List of *Caballeronia*, *Mycetohabitans*. *Mycoavidus*, *Paraburkholderia* and *Trinickia* species analyzed by MLSA and NCBI database accession numbers of the different genes.

			Gene or genome accession number		
Species	Strain	*16S*	*atpD*	*lepA*	*recA*
Caballeronia glathei	ATCC 29195	NR_037065 (strain N15)	HQ398423	HQ398519	HQ398565
Caballeronia terrestris	LMG 22937	NR_125558 (strain R-23321)		FCOL00000000.2	
Caballeronia zhejiangensis	OP-1	NR_117902		JFHD00000000.1	
***Candidatus* Xiphinematincola pachtaicus gen. nov. sp. nov.**	IAS	KT735068 KT735072	**MW485035**	**MW485036**	**MW485037**
Candidatus Glomeribacter gigasporarum	BEG34	AJ251634 (strain E28)		GCA_000227585.1	
Mycetohabitans endofungorum	HKI456	NR_042584		PRDW00000000.1	
Mycetohabitans rhizoxinica	HKI	NR_042393		FR687359	
Mycoavidus cysteinexigens	B1-EB	NR_149240		AP018150	
Paraburkholderia diazotrophica	LMG 26031	NR_117848 (strain NKMU-JPY461)		FNYE00000000.1	
Paraburkholderia kururiensis	KP23	NR_024721	HQ398439	HQ398535	HQ398581
Paraburkholderia nodosa	Br3437	NR_043181	HQ398444	HQ398539	HQ398586
Paraburkholderia phymatum	GR-01	NR_027555 (strain STM815)	HQ398448	HQ398543	HQ398590
Paraburkholderia sediminicola	LMG 24238	NR_044383 (strain HU2-65W)	HQ398452	HQ398547	HQ398594
Trinickia soli	LMG 24076	NR_043872 (strain GP25-8)	HQ398455	HQ398550	HQ398597
Outgroups					
Cupriavidus metallidurans	CH34	NR_027607		NC_0079773	
Ralstonia solanacearum	GMI1000	NR_044040		NC_003295	
